# Genetic basis of cannabis use: a systematic review

**DOI:** 10.1186/s12920-021-01035-5

**Published:** 2021-08-12

**Authors:** Alannah Hillmer, Caroul Chawar, Stephanie Sanger, Alessia D’Elia, Mehreen Butt, Raveena Kapoor, Flavio Kapczinski, Lehana Thabane, Zainab Samaan

**Affiliations:** 1 Neuroscience Graduate Program, Department of Psychiatry and Behavioural Neurosciences, McMaster University, 100 West 5th St., Hamilton, ON L8N 3K7 Canada; 2 Health Science Library, McMaster University, 1280 Main St. W., Hamilton, ON L8S 4L8 Canada; 3 Integrated Science Program, McMaster University, 1280 Main St. W., Hamilton, ON L8S 4L8 Canada; 4 Michael G. DeGroote School of Medicine, McMaster University, 1280 Main St. W., Hamilton, ON L8S 4L8 Canada; 5 Department of Health Research Method, Evidence and Impact, 1280 Main St. W., Hamilton, ON L8S 4L8 Canada

**Keywords:** Systematic review, Cannabis, Genetics, Genome-wide Association Study

## Abstract

**Background:**

With the increase in cannabis use rates, cannabis use disorder is being reported as one of the most common drug use disorders globally. Cannabis use has several known physical, psychological, and social adverse events, such as altered judgement, poor educational outcomes, and respiratory symptoms. The propensity for taking cannabis and the development of a cannabis use disorder may be genetically influenced for some individuals. Heritability estimates suggest a genetic basis for cannabis use, and several genome-wide association studies (GWASs) have identified possible regions of association, albeit with inconsistent findings. This systematic review aims to summarize the findings from GWASs investigating cannabis use and cannabis use disorder.

**Methods:**

This systematic review incorporates articles that have performed a GWAS investigating cannabis use or cannabis use disorder. MEDLINE, Web of Science, EMBASE, CINAHL, GWAS Catalog, GWAS Central, and NIH Database of Genotype and Phenotype were searched using a comprehensive search strategy. All studies were screened in duplicate, and the quality of evidence was assessed using the quality of genetic association studies (Q-Genie) tool. All studies underwent qualitative synthesis; however, quantitative analysis was not feasible.

**Results:**

Our search identified 5984 articles. Six studies met our eligibility criteria and were included in this review. All six studies reported results that met our significance threshold of *p* ≤ 1.0 × 10^–7^. In total 96 genetic variants were identified. While meta-analysis was not possible, this review identified the following genes, *ANKFN1*, *INTS7*, *PI4K2B*, *CSMD1*, *CST7*, *ACSS1*, and *SCN9A*, to be associated with cannabis use. These regions were previously reported in different mental health conditions, however not in relation to cannabis use.

**Conclusion:**

This systematic review summarized GWAS findings within the field of cannabis research. While a meta-analysis was not possible, the summary of findings serves to inform future candidate gene studies and replication efforts.

*Systematic Review Registration* PROSPERO CRD42020176016.

**Supplementary Information:**

The online version contains supplementary material available at 10.1186/s12920-021-01035-5.

## Introduction

### Rationale

Over the past two decades cannabis use and dependence are estimated to have increased, with cannabis use disorder (CUD) reported as one of the most common drug use disorders globally [1]. In Canada, it has been reported that nearly 17 percent of Canadians aged 15 years and older reported using cannabis between October and December of 2019, an increase from 14 percent between January to March of 2018. Additionally, cannabis consumption rates are higher among males than females [2]. Concerningly, cannabis has been associated with substantial adverse effects. Like other drugs, cannabis can result in cravings, dependence, and drug-seeking behaviour [3, 4]. During intoxication, cannabis can interfere with memory, motor coordination, altered judgement, and at higher doses, paranoia or psychosis [3]. Further, repeated use of cannabis can have long lasting effects, including altered brain development, poor education outcome, cognitive impairment, diminished life satisfaction and achievement, poor professional and social achievements, symptoms of chronic bronchitis and increased risk of chronic psychotic disorders [3, 5].

Heritability estimates for cannabis use initiation varied from 30 to 48%, and from 51 to 59% for problematic cannabis use, suggesting a genetic component exists [6]. Genome-wide association study (GWAS) meta-analyses have identified possible regions of association on chromosome 3 for lifetime cannabis use (*CADM2*), chromosome 10 for CUD (rs77300175), and chromosome 16 for age of first cannabis use (*ATP2C2*) [7–9]. Moreover, candidate gene studies have detected some significant associations with cannabis use on the *CNR1*, *GABRA2*, *FAAH*, and *ABCB1* genes, but as with genome-wide association studies (GWASs), replication of these associations has been inconsistent [10].

GWASs provide a ‘hypothesis-free’ method of identifying novel variant-trait associations, leading to the discovery of novel biological mechanisms and diverse clinical applications [11]. As such, in this systematic review, we will summarize GWAS findings relevant to cannabis use or CUD outcomes and discuss future directions.

Objectives:

The main goal of this systematic review is to identify genetic variants from GWASs associated with cannabis use.

Primary objectives of this systematic review include the following:

1. Identify genetic variants associated with current cannabis use. Current cannabis use is defined by either self-report or positive urine drug screens within 1 month of the study being conducted.
2. Identify genetic variants associated with lifetime cannabis use. Lifetime cannabis use is defined by any self-reported or positive urine drug screens of cannabis use within one’s lifetime.
3. Identify genetic variants associated with CUD. CUD is defined by any diagnostic and classification systems used to diagnose CUD or questionnaires validated to assess CUD.

Secondary objectives of this systematic review include the following:

1. Identify genetic variants associated with the adverse outcomes of cannabis use, including psychiatric (cognitive impairment, psychotic symptoms, depression, anxiety, suicidal behavior) and non-psychiatric (chronic bronchitis, lung infections, chronic cough, increased risk of motor vehicle accidents) [12–14].
2. When feasible, perform subgroup summaries by sex or ethnic differences.

## Methods

This systematic review is reported in accordance with the Preferred Reporting Items for Systematic Reviews and Meta-Analyses (PRISMA) statement [15] (see PRISMA checklist in Additional file 1). The Human Genome Epidemiology Network (HuGENet) guideline was used to supplement the PRISMA guideline. While this review does not conform with the HuGENet guideline expectations of reporting on candidate gene study findings, the HuGENet is used to uphold the standard of reporting research specific to genetic association studies [16].

### Protocol and registration

The protocol for this systematic review has been registered within the International Prospective Register of Systematic Reviews (PROSPERO) (registration number: CRD42020176016) [17]. The full protocol has been published in the journal of Systematic Reviews [18].

### Eligibility criteria

This review investigates GWASs presenting original data on associations between cannabis use and genetic polymorphisms using any study design (i.e. case–control, cohort, etc.). We include studies investigating CUD as well as any studies measuring any use of cannabis. Studies that investigated CUD as defined by any version of the Diagnostic and Statistical Manual (DSM) or other diagnostic and classification systems such as the International Statical Classification of Diseases and Related Health Problems-10 (ICD-10) were included. We define cannabis use based on the included studies’ definitions and accept the following definitions: current cannabis use is defined as either self-report or positive urine drug screens within one month of the study being conducted, and lifetime cannabis use is defined as any self-reported or positive urine drug screens of cannabis use within one’s lifetime [19]. All other studies that did not perform a GWAS and investigate cannabis use or CUD were excluded. No restrictions were placed on the study setting or participant’s age, sex, ethnic background or language. Further details on the inclusion criteria can be found in the study protocol [18].

### Information sources and search strategy

A Health Science Librarian was consulted to develop a comprehensive search strategy. OVID MEDLINE 1946-Present, Web of Science 1976-Present, OVID EMBASE 1974-Present, EBSCOHost CINHAL 1981-Present, GWAS Catalog, GWAS Central, and NIH Database of Genotype and Phenotype databases were searched using the established strategy, modified for each database. All databases were searched from inception to February 2^nd^, 2021. The search strategy included all terms relevant to genome-wide association studies and cannabis. The search strategies for each electronic database are provided in Table 1.

**Table 1:** Search strategy

OVID MEDLINE	1. Genome-Wide Association Study/ 2. Genotyping Techniques/ 3. Genome, Human/ 4. Genetic Variation/ 5. genetics/ or exp human genetics/ 6. (human* adj2 (genotyp* or genome* or genetic*)).ti,ab,kw,kf 7. (GWS or GWAS or GWA).mp 8. genome wide.ti,ab,kw,kf 9. 1 or 2 or 3 or 4 or 5 or 6 or 7 or 8 10. exp Cannabis/ 11. ((cannabis* or marijuana* or cannabinoids* or marihuana* or hash* or kush* or weed* or pot* or THC* or CBD*) adj2 (overdose* or use* or using or misuse* or abus* or dependence* or addict*)).ti,ab,kw,kf 12. 10 or 11 13. 9 and 12 *14. Limit 13 to humans*
Web of Science	TS = (genome-wide association study or genome-wide association or GWAS or GWA or genome wide) TS = (human NEAR/2 genome) TS = (( cannabis* or marijuana* or cannabinoids* or marihuana* or hash* or kush* or weed* or pot* or THC* or CBD*) NEAR/2 (overdose* or use* or using or misuse* or abus* or dependence* or addict*)) TS = (cannabis* or marijuana* or marihuana*) #1 OR #2 #3 OR #4 #5 and #6
OVID EMBASE	Genome-Wide Association Study/ Genotyping Techniques/ Genome, Human/ Genetic Variation/ genetics/ or exp human genetics/ (human* adj2 (genotyp* or genome* or genetic*)).ti,ab,kw. (GWS or GWAS or GWA).mp. genome wide.ti,ab,kw. 1 or 2 or 3 or 4 or 5 or 6 or 7 or 8 exp Cannabis/ ((cannabis* or marijuana* or cannabinoids* or marihuana* or hash* or kush* or weed* or pot* or THC* or CBD*) adj2 (overdose* or use* or using or misuse* or abus* or dependence* or addict*)).ti,ab,kw. 10 or 11 9 and 12 *Limit 13 to human*
EBSCOHost CINAHL	genome-wide association study or genome-wide association or GWAS or GWA or genome wide or genome cannabis* or marijuana* or cannabinoids* or marihuana* or hash* or kush* or weed* or pot* or THC* or CBD*) overdose* or use* or using or misuse* or abus* or dependence* or addict* S2 and S3 S1 and S4 *Limit to Human*
GWAS Catalog	Terms Searched: Cannabis Cannabis dependence Marihuana Marijuana Cannabinoids Hash Kush Weed Pot THC CBD
GWAS Central	Terms Searched: Cannabis Cannabis dependence Marijuana Marihuana Cannabinoids Hash Kush Weed Pot THC CBD
NIH Database of Genotypes and Phenotypes	Terms Searched: Cannabis Cannabis dependence Marijuana THC Marihuana Cannabinoids Hash Kush Weed Pot CBD

### Study selection and data collection process

Calibration was completed prior to the formal screening process. Title and abstract screening, full-text screening and data extraction phases were completed in duplicate through Covidence [20]. Conflict resolution at the title and abstract and full-text stages was performed by a senior reviewer (AH or CC), blind to the reviewer’s vote. Disagreements at the data extraction stage was resolved by the consensuses of the two reviewers. The reason for study exclusion was recorded at the full-text stage.

### Data items

Data extracted included baseline participant characteristics, the measure of cannabis used, relevant and significant measured outcomes, statistical measures, and reported study limitations and conflicts. For this review, the threshold of significance of genetic variants reaching *p* ≤ 10^–7^ was set, as some GWAS results with this significance level have been shown to be replicable within the literature [21].

### Risk of bias within studies and data analysis

Quality assessment was completed in duplicate for each included study using the Quality of Genetic Association Studies (Q-Genie) tool [Version 1.1] [22]. Disagreements of quality assessment was resolved through discussion between the two reviewers, and the first author reviewed and confirmed all quality assessments.

### Summary measures and synthesis of results

A random-effects meta-analysis through pooled odds ratios was planned to quantitatively assess the data. However, these measures were not appropriate as data extracted from each study were unique and could not be combined. For the aforementioned reasons, a heterogeneity test, and a subgroup meta-analyses could not be completed.

### Risk of bias across studies

The Grading of Recommendations Assessment, Development and Evaluation (GRADE) was used to assess the strength of evidence, with specific consideration of prognostic factors [23, 24]. GRADE scores assess outcomes according to the risk of bias, publication bias, consistency, directness, and precision [23].

## Results

### Study selection

The search strategy, along with hand-searching, yielded 5984 studies. After removing duplicates through the Zotero reference manger and Covidence, 4344 studies were unique and screened for eligibility at the title and abstract phase [20, 25]. Of the 69 studies eligible for full-text screening, 6 studies were included in this review and underwent data extraction and quality assessment.

Studies frequently failed to meet the eligibility criteria for inclusion for the following reasons (i) conducted a GWAS meta-analysis, (ii) conducted a candidate gene study or (iii) were investigating a factor associated with cannabis use (i.e. aggression) rather than cannabis use itself.

**Fig. 1:**
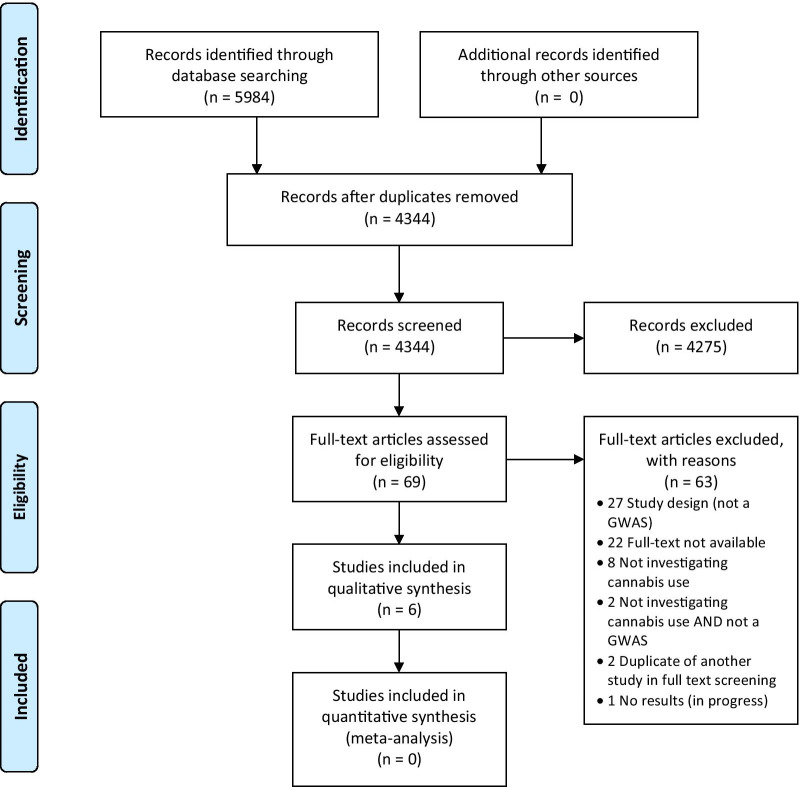
PRISMA Flow Dagram of Study Inclusion

Please see the PRISMA flow diagram in Fig. 1.

### Study characteristics

Individual study characteristics are reported in Table 2. Two studies were case–control, two were cohort, one was case-cohort, and another was case-cohort and cohort. Interestingly, the first GWAS in the field of cannabis use was published in 2011 and the most recent conducted in 2019 [26, 27]. All studies used data from large study datasets. Three studies utilized the Study of Addiction: Genetics and Environment (SAGE) [4, 26, 28]. The International Cannabis Consortium (ICC), UKBiobank, and 23andMe were utilized in one study which performed three independent GWAS on the aforementioned datasets [9]. Another study combined the Yale-Penn and the International Consortium on the Genetics of Heroin Dependence (ICGHD) to perform a single GWAS [4]. Finally, one study utilized the Integrative Psychiatric Research (iPSYCH) [27] and another the Netherlands twin registry [29]. Studies varied in size from 3053 to 51,372 participants. Of the studies which reported participants’ sex and age, three studies had a population comprised of mostly female participants [9, 26, 28, 29], while only one reported majority male [4]. The mean age of study participants varied from mid-thirties to mid-fifties. Three studies reported on participants of European or African American ethnicities [4, 26, 28] and three studies reported a European only ethnicity [9, 27, 29]. Reported outcomes of interest included lifetime cannabis use [8, 9], CUD as defined by either the DSM-IV [26] or ICD-10 [27], CUD criteria count [4, 28] or age of onset of cannabis use [29].

**Table 2:** Individual study characteristics

First Author Last Name, Year	Title of Publication	Study Design	Cohort used	Sample Size	% Male	Mean age	Ethnicity	Outcome of interest
Agrawal, 2011	A Genome-wide Association Study of DSM-IV Cannabis Dependence	Case–Control	SAGE	3054	NR	39.00	2019 European-Americans, 1035 African Americans	Life-time history of DSM-IV cannabis dependence, modified to included cannabis withdrawal
Agrawal, 2014	DSM-5 Cannabis Use Disorder: A Phenotypic and Genomic Perspective	Case–control	SAGE	3053	49%	38.10	2018 European-Americans, 1035 African Americans	DSM-5 cannabis use disorder factor scores
Demontis, 2019	Genome-wide association study implicates CHRNA2 in cannabis use disorder	Case-cohort	iPSYCH	CUD: 2387 Control: 48,985 Total: 51,372	NR	CUD: 24.77 Control: 22.67	European	International Statistical Classification of Disease and Related Health Problems, 10^th^ revision (ICD-10) diagnosis reflecting problematic and persistent use of cannabis
Minica, 2015	Heritability, SNP- and Gene-Based Analyses of Cannabis Use Initiation and Age at Onset	Cohort	Netherlands twin registry	6744	39.1%	39.09	European	Self-reported use of cannabis ever in lifetime and self-reported age of onset of cannabis use
Pasman, 2018	GWAS of lifetime cannabis use reveals new risk loci, genetic overlap with psychiatric traits, and a causal effect of schizophrenia liability	Cohort	ICC study	35,297	44.3%	35.7	European	Any cannabis use within lifetime
			UK Biobank	126,785	43.7%	55		
			23andMe	22,683	NR	NR		
Sherva, 2016	Genome-wide Association Study of Cannabis Dependence Severity, Novel Risk Variants, and Shared Genetic Risks	Cohort/Case-cohort	Yale-Penn, SAGE, ICGHD	14,754	53.4%	39.24	8754 European-American, 6000 African American	Criterion count for DSM-IV Cannabis Dependence

### Risk of bias within studies

The Q-Genie tool [version 1.1] was completed in duplicate and used to assess study quality. Studies were assessed on a scale of 1 to 7 for 11 items. An overall score greater than or equal to 45 for studies with a control group and studies with an overall score greater than 40 without a control group were considered good quality according to the Q-Genie tool [22]. All studies were considered to be good quality except for one study, Minica et al., which was deemed moderate quality. It should be noted that Minica et al. did not discuss any potential sources of bias or limitations within their study. Additionally, the study was conducted using the Netherlands twin registry and while individuals with a genetic relatedness larger than 0.025 were excluded for some analyses, heritability was not accounted for in all analyses and may therefore introduce bias [29]. Three studies reported potential conflicts of interest due to involvement with industry funding [4, 26, 28], two studies report conflict in a patent involved in identifying SNPs associated with addiction [26, 28] and one study reports authors are employees of deCODE genetics [27]. Please see Table 3 for the studies Q-genie scores of the included studies.

**Table 3:** Q-genie scores

First Author Last Name, Year	Reported conflicts of interest	Reported study limitations	Q-Genie Score	Quality Assessment
Agrawal, 2011	Drs. LJ Bierut, J. Rice, A. Goate and S Saccone are listed as inventors on the patent "Markers for Addiction" (US 20,070,258,898): covering the use of certain SNPs in determining the diagnosis, prognosis, and treatment of addiction. Dr. Bierut has acted as a consultant for Pfizer, Inc. in 2008. All other authors report no competing interests	SAGE study was ascertained for alcohol dependence led to a high level of comorbidity in the cannabis dependent cases and exposed controls Use of controls with other forms of substance dependence but not cannabis dependence protected against signals that may have been less specific Power computations revealed that minor allele frequencies ranging from 15–40% association signals with odds ration exceeding 1.45 were able to be detected	53	Good Quality
Agrawal, 2014	Laura J. Bierut is listed as an inventor on Issued U.S. Patent 8,080,371,“Markers for Addiction” covering the use of certain SNPs in determining the diagnosis, prognosis, and treatment of addiction	The sample was ascertained from three family studies of substance use disorders for the express purpose of identifying genetic variants for alcoholism, nicotine and cocaine dependence and related pathology Differences in DSM-IV and DSM-5 criteria leading to different assessments of withdrawal and diagnosis of CUD across study populations	55	Good quality
Demontis, 2019	T. Werge has been a lecturer and advisor to H. Lundbeck A/S. T.E. Thorgeirsson, D.F. Gudbjartsson, G.W. Reginsson, H. Stefansson and K. Stefansson are employees of deCODE genetics/Amgen	None reported	57	Good quality
Minica, 2015	None	No statistically significant GWAS findings that pass the threshold p < 1.0 × 10^–8^	49	Moderate quality
Pasman, 2018	P.F., S.L.E. and members of the 23andMe Research Team are employees of 23andMe Inc. J.A.R.-Q. was on the speakers’ bureau and/or acted as consultant for Eli Lilly, Janssen- Cilag, Novartis, Shire, Lundbeck, Almirall, BRAINGAZE, Sincrolab and Rubió in the last 5 years. He also received travel awards (air tickets and hotel) for taking part in psychiatric meetings from Janssen-Cilag, Rubió, Shire and Eli Lilly. The Department of Psychiatry chaired by him received unrestricted educational and research support from the following pharmaceutical companies in the last 5 years: Eli Lilly, Lundbeck, Janssen- Cilag, Actelion, Shire, Ferrer and Rubió	Lifetime cannabis was analyzed as a single dichotomous measure combining experimental and regular users in a single group The power of some analyses may have been limited	52	Good quality
Sherva, 2016	Dr Kranzler reports being a consultant or an advisory board member for Alkermes, Indivior, Lundbeck, and Otsuka (unrelated to the present study) and being a member of the American Society of Clinical Psychopharmacology’s Alcohol Clinical Trials Initiative, which is supported by AbbVie, Ethypharm, Lilly, Lundbeck, and Pfizer. No other disclosures were reported	One of the significant SNPs identified (rs143244591 on chromosome 3) has little supportive evidence for association from other SNPs in the region None of the GWAS SNPs identified in the full GWAS analysis are rare Lack of evidence of associations in both the European American and African American participants The Yale-Penn samples who underwent genotyping on the HumanOmni1-Quad and Human Core Exome chips showed more consistent results than the corresponding SAGE population The cohorts used have higher rates of polysubstance dependence than the general population and may not be generalizable to individuals who only use cannabis	52	Good quality

### Results of individual studies

All six studies included in this systematic review reported outcomes that reached the significance threshold set a priori (Table 4).

**Table 4:** SNPs reaching borderline significance threshold

First Author Last Name, Year	Outcome associated with SNP	Build	SNP IDs	Chr:Pos	Alleles	Minor Allele	Gene or Locus	MAF	N		Measure of Association type		Measure of Association value		Measure of Variability		Measure of Variability value		*p* value		Ethnicity	
Agrawal, 2011	DSM-IV cannabis dependence	hg18	rs1019238	17			ANKFN1	EA = 0.4, AA = 0.1	3054		OR		1.453		95% CI		1.254–1.682		6.1E**−**07		AA & EA	
			rs1431318	17			ANKFN1	EA = 0.44, AA = 0.25	3054		OR		0.708		95% CI		0.616–0.812		9.14E**−**07		AA & EA	
Agrawal, 2014	Cannabis use disorder factor scores from 12 DSM IV + 5 criteria	hg18	rs4364205	3		T		0.41	1035		β		− 0.19		95% CI		− 0.26− (− 0.12)		1.3E**−**07		AA	
Demontis, 2019	Cannabis use disorder	hg19	rs56372821	8	A/G	A		0.163	51,372		OR		0.728						**9.31E−12**		EA	
			rs4732724	8	C/G	C		0.324	51,372		OR		0.82						**1.34E−08**		EA	
			rs6558008	8	A/C	A		0.755	51,372		OR		1.237						**2.45E−08**		EA	
			rs73229090	8	A/C	A		0.11	51,372		OR		0.702						**7.41E−10**		EA	
			rs937220	8	A/G	A		0.259	51,372		OR		0.8258						2.46E**−**07		EA	
Minica, 2015	Cannabis initiation	GRCh37	rs35487050	19:35,221,228	C/T		ZNF181		6744		β		0.81		SE		0.16		1.68E**−**07		E	
			rs35917943	19:35,147,183	C/A			< 0.05	6744		β		0.77		SE		0.15		1.62E**−**07		E	
			rs35760174	19:35,221,582	C/G				6744		β		0.76		SE		0.15		7.04E**−**07		E	
	Age of onset of cannabis use		rs142324060	5:95,425,757	G/A			< 0.05	5148		β		0.68		SE		0.11		7.66E**−**08		E	
			rs78505392	5:95,422,966	C/G				5148		β		0.58		SE		0.1		2.16E**−**07		E	
			rs12003072	9:86,771,161	A/C				5148		β		0.52		SE		0.09		3.04E**−**07		E	
			rs77097806	5:95,456,735	A/G				5148		β		0.56		SE		0.1		3.54E**−**07		E	
			rs6879646	5:95,450,187	A/G				5148		β		0.57		SE		0.1		3.61E**−**07		E	
			rs4613744	5:95,451,494	C/T				5148		β		0.55		SE		0.1		5.07E**−**07		E	
			rs60218730	5:95,492,765	G/T				5148		β		0.59		SE		0.11		5.98E**−**07		E	
			rs74305417	9:86,779,774	C/G				5148		β		0.52		SE		0.09		6.2E**−**07		E	
			rs142981069	18:58,826,022	G/A				5148		β		0.47		SE		0.09		7.25E**−**07		E	
			rs12386084	18:58,827,145	C/G				5148		β		0.47		SE		0.09		7.25E**−**07		E	
			rs117918936	18:58,828,323	G/A				5148		β		0.47		SE		0.09		7.25E**−**07		E	
			rs2160801	18:58,829,024	T/A				5148		β		0.47		SE		0.09		7.25E**−**07		E	
			rs145424173	18:58,829,597	T/C				5148		β		0.47		SE		0.09		7.25E**−**07		E	
			rs117538409	18:58,830,942	G/C				5148		β		0.47		SE		0.09		7.25E**−**07		E	
			rs17817245	18:58,832,135	A/G				5148		β		0.47		SE		0.09		7.25E**−**07		E	
			rs140206809	18:58,833,215	A/G				5148		β		0.47		SE		0.09		7.25E**−**07		E	
			rs117692712	18:58,834,506	T/G				5148		β		0.47		SE		0.09		7.25E**−**07		E	
			rs17817423	18:58,835,462	C/T				5148		β		0.47		SE		0.09		7.25E**−**07		E	
			rs9916935	18:58,835,931	T/C				5148		β		0.47		SE		0.09		7.25E**−**07		E	
			rs192013604	18:58,838,324	T/C				5148		β		0.47		SE		0.09		7.25E**−**07		E	
			rs117471640	18:58,838,402	A/G				5148		β		0.47		SE		0.09		7.25E**−**07		E	
			rs78456402	9:86,781,900	C/A				5148		β		0.5		SE		0.09		9.09E**−**07		E	
			rs11998981	9:86,783,107	T/C				5148		β		0.5		SE		0.09		9.09E**−**07		E	
			rs79236058	5:95,478,830	G/A				5148		β		0.57		SE		0.1		9.59E**−**07		E	
Pasman, 2018	Lifetime cannabis use	hg19	rs7609594	3:85,482,595	G/A	A	CADM2	0.38	126,785		β		0.068		SE		0.01		**5.86E−11**		E	
			rs4099556	4:37,067,936	G/A	A	MIR4801	0.176	126,785		β		−0.079		SE		0.013		**3.56E−09**		E	
			rs78680891	3:51,028,954	C/G	C		0.002299	126,574		β		− 0.52473		SE		0.106473		8.30E**−**07		E	
			rs2071704	4:3,240,159	C/T	C		0.419929	125,953		β		− 0.04974		SE		0.010162		9.83E**−**07		E	
			rs76565656	7:148,048,453	G/A	G		0.122539	124,918		β		− 0.07974		SE		0.015258		1.73E**−**07		E	
			rs4308708	8:76,702,058	A/C	A		0.041699	126,193		β		− 0.13628		SE		0.025107		5.70E**−**08		E	
			rs10883796	10:104,655,315	G/A	G		0.296441	126,444		β		− 0.05638		SE		0.010951		2.62E**−**07		E	
			rs11186071	10:91,969,337	G/A	G		0.004168	126,711		β		− 0.90907		SE		0.18524		9.22E**−**07		E	
			rs11214441	11:112,846,713	T/A	T		0.394257	126,761		β		− 0.05354		SE		0.010263		1.82E**−**07		E	
			rs5442	12:6,954,864	G/A	G		0.066921	126,785		β		− 0.09622		SE		0.019181		5.27E**−**07		E	
			rs75448266	12:6,798,632	A/C	A		0.084049	125,672		β		− 0.0944		SE		0.017746		1.04E**−**07		E	
			rs17083392	13:69,026,559	A/G	A		0.004286	126,745		β		− 1.60924		SE		0.3177		4.08E**−**07		E	
			rs2019135	13:69,034,323	T/C	T		0.004267	126,772		β		− 1.76061		SE		0.323766		5.39E**−**08		E	
			rs2019150	13:69,034,188	C/A	C		0.004262	126,771		β		− 1.76061		SE		0.323766		5.39E**−**08		E	
			rs677755	13:69,041,438	T/C	T		0.00486	126,760		β		1.426		SE		0.290601		9.24E**−**07		E	
			rs74632168	13:69,048,954	C/T	C		0.004056	126,772		β		− 1.72062		SE		0.319014		6.91E**−**08		E	
			rs2650494	16:28,318,440	A/G	A		0.414312	124,286		β		− 0.05259		SE		0.010223		2.68E**−**07		E	
			rs325363	18:38,331,295	G/A	G		0.229965	126,624		β		0.059212		SE		0.011689		4.07E**−**07		E	
			rs4492854	11:112,983,534	C/T	C		0.4363	30,366		β		0.1113		SE		0.021251		1.63E**−**07		E	
Sherva, 2016	DSM-IV cannabis dependence criteria count	GRCh37	-	1:212,183,114:I			INTS7		1250										2.05E**−**07		AA	
			rs141482228				INTS7		1250										**8.84E−10**		AA	
			rs77349458				SNORA26,INTS7		1250										1.57E**−**07		AA	
			rs77448142				RPS20P10,RPS20P10-CYP26B1		1250										2.13E**−**07		AA	
			-	2:167,239,818:D			SCN9A-SCN7A		4750										7.43E**−**07		AA	
			rs313542				PI4K2B		2640										**7.12E−10**		EA	
			rs7689780				PI4K2B,PI4K2B-ZCCHC4		2640										4.63E**−**07		EA	
			rs73323306				ARL2BPP5-RP11-541P9.3		4750										**7.61E−09**		AA	
			rs7832545				CSMD1		2640										**3.61E−10**		EA	
																			3.02E**−**07*			
			rs77378271				CSMD1		2640										**5.30E−10**		EA	
																			6.69E**−**07*			
			rs75721860				CSMD1		2640										**2.75E−09**		EA	
			rs7853028				PSMB7		1250										**8.94E−09**		AA	
			rs4643011				HABP2		1250										7.21E**−**07		AA	
			rs116474042				RP11-755E23.3-CCDC67		4750										3.81E**−**07		AA	
			rs189167038				SNORD11-RNU6-1014P		1250										**2.64E−09**		AA	
			rs142162415				SNORD11-RNU6-1014P		1250										**1.88E−08**		AA	
			rs117047810				SNORD11-RNU6-1014P		1250										5.68E**−**08		AA	
			rs11466037				MEFV		1250										8.08E**−**07		AA	
			rs183568578				MEFV		1250										5.62E**−**07		AA	
			rs114269992				RP5-860P4.2-CST7		1250										2.13E**−**07		AA	
																			1.63E**−**07*			
			rs114620529				RP5-860P4.2-CST7		1250										2.15E**−**07 1.61E**−**07		AA	
			rs116629084				RP5-860P4.2-CST7		1250										1.70E**−**07		AA	
																			1.27E**−**07*			
			rs115384512				RP5-860P4.2-CST7		1250										8.91E**−**08		AA	
																			7.19E**−**08*			
			rs147641662				CST7,RP5-860P4.2-CST7		1250										8.87E**−**08		AA	
																			7.13E**−**08*			
			rs191783144				CST7,RP5-860P4.2-CST7		1250										8.81E**−**08		AA	
																			7.07E**−**08*			
			rs146806338				CST7,RP5-860P4.2-CST7		1250										8.80E**−**08		AA	
																			7.03E**−**08*			
			rs114828727				CST7,APMAP,CST7-APMAP		1250										9.01E**−**08		AA	
																			6.92E**−**08*			
			rs114128002				APMAP		1250										8.06E**−**08		AA	
																			6.33E**−**08*			
			rs115342711				RNU6-1257P,APMAP		1250										2.54E**−**07		AA	
																			1.15E**−**07*			
			-	20:24,955,422:I			RNU6-1257P,APMAP		1250										2.54E**−**07		AA	
																			1.15E**−**07*			
			rs115764440				APMAP		1250										2.33E**−**07		AA	
																			9.03E**−**08*			
			rs116068335				APMAP-ACSS1		1250										1.66E**−**07		AA	
																			5.62E**−**08*			
			rs114637142				ACSS1,APMAP-ACSS1		1250										9.15E**−**08		AA	
			rs114071901				ACSS1		1250										1.06E**−**07		AA	
			rs113232742				ACSS1		1250										8.82E**−**08		AA	
			rs114199928				ACSS1		1250										8.42E**−**08		AA	
			rs114836364				ACSS1		1250										8.23E**−**08		AA	
			rs116669368				ACSS1		1250										5.08E**−**08		AA	
			rs145379934				ACSS1		1250										**3.96E−08**		AA	
			rs143020225	2:167,214,714	G/A	G	snoU13,SCN9A	0.95	4750										5.85E**−**07		AA	
			-	chr22:26,917,069:I			C9.15,TPST2,CTA-445C9.14-CTA-4		2640										**3.15E−10**		EA	
			rs4149485				TPST2,CTA-445C9.15		2640										**2.24E−10**		EA	

^*^Adjusted for the DSM-IV criteria counts for alcohol, cocaine, and opioid dependence

AA = African American, EA = European American, E = European

Bold type indicates variants for which the p value reached genome-wide significance (*p* ≤ 5 × 10^–8^)

Agrawal et al. [26] identified two SNPs associated with DSM-IV cannabis dependence within the *ANKFN1* gene (chromosome 17). European and African American participants were selected from the SAGE study which was aimed to primarily study DSM-IV alcohol dependence. Case status was defined as a lifetime history of DSM-IV cannabis dependence, with controls defined as using cannabis at least once in their lifetime but not meeting criteria for DSM-IV cannabis dependence.

Agrawal et al. [28] identified a SNP reaching borderline significance threshold on chromosome 3 associated with CUD factor scores in African Americans, however, no associated gene was identified. Participants were European and African Americans selected from the SAGE study. DSM factor scores were developed from 12 DSM-IV and DSM-5 criteria for CUD.

Demontis et al. identified 26 SNPs associated with CUD on chromosome 8, with no associated gene identified. However, only 5 SNPs were discussed and identified in the paper, and thus only 5 SNPs are reported in this review. Participants were selected from the iPSYCH cohort and were of European ancestry. The iPSYCH cohort was established to study six major psychiatric disorders, however, identified participants meeting ICD-10 CUD [27].

Minica et al. reported 3 SNPs associated with cannabis initiation and 24 SNPs associated with the age of onset of cannabis use. Identified SNPs were found on chromosomes 5, 9, 18 and 19, with one SNP associated with cannabis initiation was found on the Zinc finger protein, *ZNF181*. All participants were of European descent and were selected from the Netherlands Twin Registry. Cannabis initiation was defined as ever/never having used cannabis while age of onset was determined by asking participants an open-ended question [29].

Pasman et al. conducted three independent GWASs in three separate cohorts, all of which included European participants: ICC, UKBiobank, and 23andMe. While results from 23andMe were unable to be shared due to privacy policies, the lead author kindly provided SNPs reaching borderline significance threshold with lifetime cannabis use for GWAS conducted in the ICC and UKBiobank cohort. One SNP in the ICC cohort and 18 SNPs in the UKBiobank were associated with lifetime cannabis use, with no genes specified in either. Lifetime cannabis use was defined as any cannabis use during lifetime [9].

Sherva et al. identified 42 SNPs associated with DSM-IV cannabis dependence criteria count across 27 different genes/regions including *INTS7, SNORA26, RPS20P10, PI4K2B, CSMD1, PSMB7, HABP2, MEFV, CST7, APMAP, ACSS1, snoU13, TPST2, SCN9A, CTA-445C9.15, CTA-445C9.14-CTA-4, SCN9A-SCN7A, ARL2BPP5-RP11-541P9.3, RP11-755E23.3-CCDC67, SNORD11-RNU6-1014P, RP5-860P4.2-CST7, RNU6-1257P, APMAP-ACSS1, C9.15, RPS20P10-CYP26B1, PI4K2B-ZCCHC4*, and *CST7-APMAP*. European and African American participants were selected from the Yale-Penn Study, the SAGE study and the ICGHD cohorts [4].

While no SNPs were reported within the same region not allowing further quantitative analysis, several phenotypic similarities exist across studies. Interestingly, two studies found that educational attainment was negatively associated with CUD [26, 27] and a third found positive genetic correlations with educational attainment [9]. Two studies found that cannabis dependence was significantly related to alcohol, nicotine, and cocaine dependence [4, 26] with a third reporting a positive genetic correlation between lifetime cannabis use and smoking and alcohol use and dependence [9].

### Risk of bias across studies

Outcomes assessed for GRADE include lifetime cannabis use, diagnosis of CUD, criterion count for CUD and age of onset of cannabis use. All outcomes included two studies except for age of onset of cannabis use which only included one study. The full GRADE table can be found in Table 5. All outcomes were rates as important, and no outcome was rated as having a “very serious” concern pertaining to any certainty criteria. Only the outcomes of diagnosis of CUD and criterion count for CUD had a serious rating, both of which were in the category of indirectness. Both of these outcomes were downgraded due to the use of different diagnostic criteria. More specifically, for the outcome of diagnosis of CUD Agrawal et al. [26] utilized the DSM-IV and Demontis et al. (2019) utilized the ICD-10 and for the outcome of criterion count of CUD Sherva et al. [4] utilized the DSM-IV criteria and Agrawal et al. [28] utilized a combination of DSM-IV and DSM-5 criteria.

**Table 5:** GRADE Assessment

No of studies	Certainty assessment						Effect			Certainty	Importance
	Study design	Risk of bias	Inconsistency	Indirectness	Imprecision	Other considerations	No of events	No of individuals	Rate (95% CI)		
**Cannabis use in lifetime**											
2	Observational studies	Not serious	Not serious	Not serious	Not serious					–	IMPORTANT
**Diagnosis of cannabis use disorder**											
2	Observational studies	Not serious	Not serious	Serious ^a^	Not serious					–	IMPORTANT
**Criterion count of cannabis use disorder**											
2	Observational studies	Not serious	Not serious	Serious ^a^	Not serious		–			–	IMPORTANT
**Age of onset of cannabis use**											
1	Observational studies	Not serious	Not serious	Not serious	Not serious		–			–	IMPORTANT

^a^Different diagnostic classification

## Discussion

### Summary of evidence

In this review we identified 96 genetic variants to be associated with different measures of cannabis. Of these genetic variants, 18 reached the genome-wide significance threshold of *p* ≤ 5 × 10^–8^, all of which are available in Table 4. As no genetic variants included in this review were reported in more than one study, meta-analyses were not possible. However, of the genetic variants identified in this review, several are located on genes in which previous studies have reported associations with mental health, namely *ANKFN1*, *INTS7*, *PI4K2B*, *CSMD1*, *CST7*, *ACSS1*, and *SCN9A*.

With cannabis being a legal substance, research on the benefits and harms of cannabis has been on the rise. However, a limited number of GWASs have been conducted on cannabis use to determine any genetic associations. This systematic review was able to qualitatively summarize findings from GWASs reporting borderline genome-wide significance to aid in identifying SNPs that may be replicable in future studies. We have identified six eligible studies that reported independent GWAS results, one of which primarily focused on a GWAS meta-analysis. Of the included studies, only participants from European or African American ethnicities were included, suggesting a need for genetic studies being conducted in more diverse ethnic populations. All six studies reported at least one borderline significant SNP; however, no two studies identified the same SNP. SNPs were found to be associated with CUD, cannabis initiation, age of onset of cannabis use, DSM-IV cannabis dependence criteria count, or lifetime cannabis use on various gene regions. According to assessment using the Q-genie tool and GRADE tool, no study or outcome was deemed to be of poor quality. Additionally, with GWAS requiring a sample size of thousands of participants for adequate power, all studies met this threshold [30].

While the majority of genes identified in the included studies had either no known function or biological plausibility, and none had any additional associations with cannabis use, as mentioned above, several did have associations with mental health conditions and are discussed briefly, namely *ANKFN1*, *INTS7*, *PI4K2B*, *CSMD1*, *CST7*, *ACSS1*, and *SCN9A*. *ANKFN1* is a protein coding gene which has been associated with smoking cessation and nicotine dependence [31]. *INTS7* is a component of the integrator complex, which is involved in the small nuclear RNA U1 and U2 transcriptions [32] and has been associated with bipolar temperament [31, 33]. *PI4K2B* contributes to the overall PI4-kinase activity of the cell [32] and is associated with attention deficit hyperactivity disorder (ADHD), logical memory and abnormality of neuronal migration [33]. *CSMD1* has been associated with behavioural disinhibition, schizophrenia, cognitive tests, chronic bronchitis, and bipolar disorder [31, 33]. *CST7* is associated with alcohol consumption and myocardial infarction [31, 33]. *ACSS1* catalyzes the synthesis of acetyl-CoA and has been associated with performance on standardized cognitive tests and bitter alcoholic beverage consumption [31–33]. *SCN9A* medicates the voltage-dependent sodium ion permeability of excitable membranes and plays a role in pain mechanisms, especially in the development of inflammatory pain [31]. As it is known that cannabis can have a negative impact on learning, memory and chronic bronchitis, known relation to mental illness and suggested role in pain management, these regions may have implications in cannabis use despite having no clear known biological relevance [3, 19].

Additionally, it is also important to highlight that genes identified in this review associated with cannabis use or CUD have also been associated with other neuropsychiatric disorders namely nicotine dependence, ADHD, bipolar disorder, schizophrenia, and alcohol consumption suggesting that the genetic risk for the development of these disorders may not be independent. Previously genetic associations have been found amongst schizophrenia, bipolar disorder, ADHD, depression, and autism spectrum disorder, with a high genetic correlation between schizophrenia and bipolar disorder and a moderate correlation between ADHD and depression, ADHD and autism spectrum disorder, and ADHD and depression [34]. A recent GWAS meta-analysis added to the evidence on shared genetic associations amongst neuropsychiatric disorders by identifying that an increased risk of cannabis use disorder is genetically correlated with increased liability for smoking initiation, alcohol use, nicotine dependence, and psychiatric disorders (e.g. ADHD, schizophrenia, major depression) [35]. These genetic correlations among neuropsychiatric disorders, including cannabis, could reflect genuine pleiotropy or could indicate these psychiatric disorders, including CUD, are not completely independent [34, 35]. As such, it is important to discuss the biological and individual factors that influence the development of neuropsychiatric disorders.

Neuropsychiatric disorders are influenced by a range of factors, including genetics, personality/mood characteristics, psychological status, behaviour, neurocognitive functioning, and demographic characteristics [36, 37]. To begin, non-specific to CUD, the prenatal environment, including prenatal nutrition, maternal stress, and maternal substance abuse, can impact brain development and therefore the behavioural outcome of children. Potential mechanisms through which the prenatal environment can impact brain development occurs on multiple levels including genetic selection, epigenetic modification, mediation of brain-immune communications, abnormal metabolism pathways, synthetic mediation of hormones and the hypothalamic–pituitary–adrenal axis, and mediation of the microbiota-gut brain axis [37]. Furthermore, nutritional deficiency during critical stages of pregnancy has been linked to emotional and behavioural problems in children including decreased attention, decreased IQ, language delay, and neurodevelopment and related neuropsychiatric disorders [37–39]. More specifically, prenatal malnutrition has been linked to an increased risk of schizophrenia during the 1944–1945 Dutch Hunger Winter and the 1959–1961 Chinese famine. Additionally, a “U” relationship between serum 12(OH)D concentration and emotion, behaviour and attention has been found [38, 40]. Interestingly, the hippocampus, which plays an important role in learning and memory, has been suggested to be sensitive to the exposure of prenatal nutrition deficiency [39, 41]. The hippocampus has also been proven to be crucial in the pathophysiology of many neuropsychiatric disorders, in which the changes result from alerted brain development [41]. Maternal stress has been associated with poor offspring outcomes including cognition, health and educational attainment, however methodological challenges exist leading to potential misattribution of socially mediated (i.e. postnatal parenting) mechanisms to biological ones (i.e. alterations to developing fetal brain) [42, 43]. Finally, prenatal exposure to alcohol and other substances has been increasingly common and the consequence of the exposure differs depending on the substance used. Alcohol, tobacco, cannabis and opioids are among the most frequent used substances during pregnancy and offspring outcomes may include birth defects, developmental disability, fetal alcohol syndrome, childhood obesity, decreased birth weight, poor inhibitory control and other organ deficits [44]. Thus, many neuropsychiatric disorders appear to result from interactions among genetic background, the prenatal environment and postnatal lifestyle choices [45, 46]. Given the known association between deficits within the prenatal environment and other neuropsychiatric disorders it is plausible to suggest that the prenatal environment and subsequential gene expression may play a role in future cannabis use and/or CUD.

As previously mentioned, a variety of factors contribute to the complex etiology of neuropsychiatric disorders such as epigenetic modification. Epigenetic modifications that can regulate gene expression include DNA methylation, nucleosomal structure and positioning, post-translational modification of nucleosome histones, histone replacement and small RNA molecules that influence protein production [47]. The most studied form of epigenetic modification is DNA methylation, which can be influenced by a range of factors including genetic factors, disease, environmental exposures, and lifestyle. DNA methylation changes can be either persistent or reversible once the exposure is no longer present, adding value for biomarker development [48]. How cannabis, THC and other exogenous cannabis receptor modulators alter epigenetic mechanisms have been previously reviewed [47]. Relatively little is known about the molecular pathways influenced by cannabis, however, one study identified 13 proteins, 3 metabolites and 2 lipids significantly associated with a metabolite of THC and another found acute effects of cannabis or THC on the central nervous system and heart rate [49, 50]. In addition to DNA methylation, post-transcriptional chemical medication of RNA is rapidly emerging as a key role in regulating gene expression, known as epitranscriptomics [51]. Of growing interest within this felid is N4-acetylcytidine (ac4C), a key role in the transcriptional translation process. ac4C has been implicated in the occurrence of various disease such as inflammation, metabolic diseases, autoimmune diseases, and cancer [52]. While the role ac4C may play in neuropsychiatric disorders remains unknown, it is important to consider the role epitransciptomics plays in the gene expression with normal development.

Current knowledge on cannabis has demonstrated that cannabis can induce structural changes to brain regions including the hippocampus, amygdala, cerebellum, prefrontal cortex and striatum as well as grey matter volume [53–55]. Potential pre-existing neurobiological factors may exist in cannabis use as well as gene x drug interactions. For instance, in young teens, reduced orbitofrontal cortex volume has been found to predict initiation of cannabis use in later adolescence. The G allele of rs2023239 of *CNR1* is linked with higher cortical CBR1 and is associated with smaller hippocampal volume in chronic cannabis users, but not healthy controls and findings that suggest only individuals with a high genetic risk of schizophrenia experience a negative impact on cortical maturation during early adolescence thus suggestive of gene × drug interactions [56–58]. In addition, functional MRI evidence suggest specific brain activity signatures with cannabis use such as increased functional connectivity associated with the default node network and insula networks and hippocampal and parahippocampal atrophy have been associated with chronic cannabis use [59, 60]. However, neuroimaging studies of cannabis users have yielded inconsistent findings and may reflect individual differences that preceded cannabis use. The inconsistent findings in the literature highlight the need for large longitudinal studies utilizing before-and-after cannabis use neuroimaging [61]. Taken together, it is plausible that structural differences in brain regions could be influenced by genetic differences between individuals, explaining the mixed evidence within neuroimaging. Further research is required to determine the complex interactions amongst individual genetic predispositions, prenatal environment, and postnatal environment contributing to individual cannabis use behaviour and/or the development of CUD. Understanding the genetic predispositions is one piece of the puzzle in understanding the complex development of cannabis use and CUD.

Finally, it is important to consider the shared genetic basis of other substance use disorders. Heritability estimates across substance use disorders vary, with heritability lowest for hallucinogens (0.39) and highest for cocaine use (0.72) [62, 63]. Additionally, substance use disorders are the result of gene x environment interactions, with partial risk inborn and another part determined by environmental experiences [62]. Previous reviews have summarized the literature on GWASs for various substance use disorders including alcohol use disorder, nicotine use disorder, CUD, OUD, and cocaine use disorder. However, genetic studies within specific substance use disorders have had varying success in replicating previously identified associations, limiting evidence for shared genetic basis across substance use disorders [63, 64]. The complexity of substance use disorder make genetic prediction efforts difficult, and while currently only alcohol use disorder have been genetically correlated with CUD, continued advancements in molecular genetic studies and substance use disorder at larges further our understanding of the biological pathways underlying substance use disorders [9, 63, 65]. For instance, *CNR1* and *CNR2*, components of the endocannabinoid system, are major targets of investigation for their impact in neuropsychiatry and addiction phenotypes suggested shared genetic risk factors [66, 67]. In regards to neuropsychiatric disorders, Mendelian randomization studies have found mixed evidence on the causal effect of cannabis initiation and schizophrenia, finding weak evidence that cannabis initiation increases schizophrenia risk and strong evidence that schizophrenia liability increases the odds of cannabis initiation, and causal evidence of ADHD on cannabis initiation [68–72]. Through continued advances, it is hoped that the underlying genetic basis for CUD, or a shared genetic basis for all substance use disorders, will be identified to provide preventative measures and treatment for substance use disorders in the future.

### Limitations

While this systematic review was rigorous and involved a peer-reviewed protocol, it is not without limitations. First, our inclusion criteria limited our review to only GWASs, meaning any GWAS meta-analyses and candidate gene studies were excluded. GWAS meta-analyses and candidate gene studies are often more powered due to their larger sample sizes and minimal genetic variants tested, respectfully [11]. However, including only GWASs was decided a priori to capture novel genetic variants associated with cannabis use and avoid the inclusion of multiple studies which could use the same genetic dataset. Second, it is important to note that this review is susceptible to publication bias, as studies that do not achieve genome-wide significance may be less likely to be published, and thus, not included in this review. Unpublished GWAS findings may exist with SNPs reaching the borderline significance threshold. While we cannot eliminate publication bias entirely, we searched abstracts, GWAS catalogs, and databases for any near significant findings that were not published. If a relevant abstract was identified, without the full study published, the first author was contacted to determine whether the full GWAS had been published or was going to be submitted to a journal. Finally, if a study met our inclusion criteria but did not report any SNPs that fell below the genome-wide significance threshold, study authors were contacted to confirm if any SNPs had reached the borderline significant threshold set for this review. Third, due to the heterogeneity of the reported findings, it was not possible to conduct a meta-analysis or sex and ethnicity subgroup analyses. Although we could not conduct a meta-analysis, we qualitatively summarized the studies and reported a comprehensive list of all SNPs reaching the significance threshold for this study.

## Conclusion

This systematic review was able to summarize GWAS findings within the field of cannabis use. The results can inform future candidate gene studies and GWASs of possible replicable SNPs that require further investigation. We were able to identify all GWASs conducted on cannabis use, highlighting the need for further research as no two GWASs reported the same SNP or gene associated with cannabis use. Further, included GWASs had limited ethnic diversity, with only European or African American participants. Recommendations are made for future research to replicate reported associations and include diverse ethnic populations to test whether SNPs associated with cannabis use reported are generalizable across study populations and if associations differ by ethnicity.

## Supplementary Information

**Additional File 1**. PRISMA 2009 checklist.

## Data Availability

All data generated or analysed during this study are included in this published article. Primary articles included in this systematic review can be found at the following links: https://onlinelibrary.wiley.com/doi/abs/10.1111/j.1369-1600.2010.00255.x. https://www.sciencedirect.com/science/article/abs/pii/S0376871613004766. https://www.nature.com/articles/s41593-019-0416-1. https://link.springer.com/article/10.1007/s10519-015-9723-9. https://www.nature.com/articles/s41593-018-0206-1?fbclid=IwAR20cNM7ZDBKTGp7QNtm521tkEECrKyDTFLQ5pQV9emvsIbrsM_TMeAUTSg. https://jamanetwork.com/journals/jamapsychiatry/article-abstract/2504223.

## Abbreviations

GWAS: Genome-wide Association Study
CUD: Cannabis Use Disorder
GWASs: Genome-wide Association Studies
PRISMA: Preferred Reporting Items for Systematic Reviews and Meta-Analyses
HuGeNET: The Human Genome Epidemiology Network
PROSPERO: International Prospective Register of Systematic Reviews
DSM: Diagnostic and Statistical Manual
ICD-10: International Statistical Classification of Diseases and Related Health Problems -10
Q-Genie: The quality of genetic association studies
SAGE: Study of Addiction: Genetics and Environment
ICC: International Cannabis Consortium
ICGHD: International Consortium on the Genetics of Heroin Dependence
iPSYCH: Integrative Psychiatric Research
ADHD: Attention-Deficit Hyperactivity Disorder

## References

[1] Degenhardt L, Charlson F, Ferrari A, Santomauro D, Erskine H, Mantilla-Herrara A (2018). The global burden of disease attributable to alcohol and drug use in 195 countries and territories, 1990–2016: a systematic analysis for the Global Burden of Disease Study 2016. Lancet Psychiatry.

[2] Statistics Canada. National Cannabis Survey, forth quarter 2019. 2019.

[3] Volkow ND, Baler RD, Compton WM, Weiss SRB (2014). Adverse health effects of Marijuana use. N Engl J Med.

[4] Sherva R, Wang Q, Kranzler H, Zhao H, Koesterer R, Herman A (2016). Genome-wide association study of cannabis dependence severity, novel risk variants, and shared genetic risks. JAMA Psychiat.

[5] Bahji A, Stephenson C (2019). International perspectives on the implications of cannabis legalization: a systematic review & thematic analysis. Int J Environ Res Public Health.

[6] Verweij KJH, Zietsch BP, Lynskey MT, Medland SE, Neale MC, Martin NG (2010). Genetic and environmental influences on cannabis use initiation and problematic use: a meta-analysis of twin studies. Addiction.

[7] Agrawal A, Chou YL, Carey CE, Baranger DAA, Zhang B, Sherva R (2018). Genome-wide association study identifies a novel locus for cannabis dependence. Mol Psychiatry.

[8] Minică CC, Verweij KJH, Most PJ, Mbarek H, Bernard M, Eijk KR (2018). Genome-wide association meta-analysis of age at first cannabis use. Addiction.

[9] Pasman JA, Verweij KJH, Gerring Z, Stringer S, Sanchez-Roige S, Treur JL (2018). GWAS of lifetime cannabis use reveals new risk loci, genetic overlap with psychiatric traits, and a causal influence of schizophrenia. Nat Neurosci.

[10] Stringer S, Minică CC, Verweij KJH, Mbarek H, Bernard M, Derringer J (2016). Genome-wide association study of lifetime cannabis use based on a large meta-analytic sample of 32 330 subjects from the International Cannabis Consortium. Transl Psychiatry.

[11] Tam V, Patel N, Turcotte M, Bossé Y, Paré G, Meyre D (2019). Benefits and limitations of genome-wide association studies. Nat Rev Genet.

[12] The Government of Canada. Cannabis Health Effects. 2019.

[13] Hall W (2015). What has research over the past two decades revealed about the adverse health effects of recreational cannabis use?. Addiction.

[14] Gobbi G, Atkin T, Zytynski T, Wang S, Askari S, Boruff J (2019). Association of cannabis use in adolescence and risk of depression, anxiety, and suicidality in young adulthood: a systematic review and meta-analysis. JAMA Psychiat.

[15] Moher D, Liberati A, Tetzlaff J, Altman D, Group P, The PRISMA Group. Preferred reporting items for systematic reviews and meta-analyses: The PRISMA Statement. PLoS Med. 2009;6(7):e1000097. 10.1371/journal.pmed.1000097 PMC2707599 19621072

[16] Little J, Higgins JPT, Bray M, Ioannidis J, Khoury M, Manolio T, et al. The HuGENet^TM^ HuGE review handbook, version 1.0. Ottawa, Ontario, Canada HuGENet Canada Coord Cent. 2006;

[17] Hillmer A, Chawar C, D’Elia A, Kapoor R, Butt M, Samaan Z. A systematic review and meta-anlaysis of genome-wide association studies and cannabis use. PROSPERO: International prospective register of systematic reivews. 2020.

[18] Hillmer A, Chawar C, Sanger S, D’Elia A, Butt M, Kapoor R (2020). Genetic determinants of cannabis use: a systematic review protocol. Syst Rev.

[19] National Academies of Sciences Engineering and Medicine. The health effects of cannabis and cannabinoids: The current state of evidence and recommendations for research [Internet]. Washington, DC: National Academies Press; 2017. Available from: https://www.ncbi.nlm.nih.gov/books/NBK423845/. 10.17226/24625 28182367

[20] Veritas Health Innovation. Covidence systematic review software [Internet]. Melbourne, Australia; Available from: https://www.covidence.org/

[21] Dudbridge F, Gusnanto A (2008). Estimation of significance thresholds for genomewide association scans. Genet Epidemiol Off Publ Int Genet Epidemiol Soc.

[22] Sohani ZN, Meyre D, de Souza RJ, Joseph PG, Gandhi M, Dennis BB, et al. Assessing the quality of published genetic association studies in meta-analyses: The quality of genetic studies (Q-Genie) tool. BMC Genet. 2015;16. 10.1186/s12863-015-0211-2 PMC4431044 25975208

[23] Guyatt GH, Oxman AD, Schünemann H, Tugwell P, Knottnerus A (2011). GRADE guidelines: a new series of articles in the Journal of Clinical Epidemiology. J Clin Epidemiol.

[24] Foroutan F, Guyatt G, Zuk V, Vandvik PO, Alba AC, Mustafa R (2020). GRADE Guidelines 28: Use of GRADE for the assessment of evidence about prognostic factors: rating certainty in identification of groups of patients with different absolute risks. J Clin Epidemiol.

[25] Center for History New Media. Zotero: The Next-Generation Research Tool [Internet]. George Mason University; 2009. Available from: http://www.zotero.org/

[26] Agrawal A, Lynskey MT, Hinrichs A, Grucza R, Saccone SF, Krueger R (2011). A genome-wide association study of DSM-IV cannabis dependence. Addict Biol.

[27] Demontis D, Rajagopal VM, Thorgeirsson TE, Als TD, Grove J, Leppälä K (2019). Genome-wide association study implicates CHRNA2 in cannabis use disorder. Nat Neurosci.

[28] Agrawal A, Lynskey MT, Bucholz KK, Kapoor M, Almasy L, Dick DM (2014). DSM-5 cannabis use disorder: a phenotypic and genomic perspective. Drug Alcohol Depend.

[29] Minică CC, Dolan CV, Hottenga J-J, Pool R, Fedko IO, Mbarek H (2015). Heritability, SNP-and gene-based analyses of cannabis use initiation and age at onset. Behav Genet.

[30] Ziyatdinov A, Kim J, Prokopenko D, Privé F, Laporte F, Loh P-R, et al. Estimating the effective sample size in association studies of quantitative traits. bioRxiv. 2019; 10.1093/g3journal/jkab057 PMC8495748 33734375

[31] Bethesda (MD): National Library of Medicine (US) National Centre for Biotechnology Infromation. dbGene [Internet]. 2004. Available from: https://www.ncbi.nlm.nih.gov/gene/

[32] UniProt Consortium (2019). UniProt: a worldwide hub of protien knowledge. Nucleic Acids Res.

[33] Yates AD, Achuthan P, Akanni W, Allen J, Allen J, Alvarez-Jarreta J (2020). Ensembl 2020. Nucleic Acids Res.

[34] Wu Y, Cao H, Baranova A, Huang H, Li S, Cai L (2020). Multi-trait analysis for genome-wide association study of five psychiatric disorders. Transl Psychiatry.

[35] Johnson EC, Demontis D, Thorgeirsson TE, Walters RK, Polimanti R, Hatoum AS (2020). A large-scale genome-wide association study meta-analysis of cannabis use disorder. Lancet Psychiatry.

[36] Taylor M, Cousijn J, Filbey F (2019). Determining risks for cannabis use disorder in the face of changing legal policies. Curr Addict Rep.

[37] Yan X, Zhao X, Li J, He L, Xu M (2018). Effects of early-life malnutrition on neurodevelopment and neuropsychiatric disorders and the potential mechanisms. Prog Neuro-Psychopharmacol Biol Psychiatry.

[38] Wang X, Jiao X, Xu M, Wang B, Li J, Yang F (2020). Effects of circulating vitamin D concentrations on emotion, behavior and attention: a cross-sectional study in preschool children with follow-up behavior experiments in juvenile mice. J Affect Disord.

[39] Chen J, Zhao X, Cui L, He G, Wang X, Wang F (2020). Genetic regulatory subnetworks and key regulating genes in rat hippocampus perturbed by prenatal malnutrition: implications for major brain disorders. Aging (Albany NY).

[40] Xu M-Q, Sun W-S, Liu B-X, Feng G-Y, Yu L, Yang L (2009). Prenatal malnutrition and adult schizophrenia: further evidence from the 1959–1961 Chinese famine. Schizophr Bull.

[41] Oberlander VC, Xu X, Chini M, Hanganu-Opatz IL (2019). Developmental dysfunction of prefrontal–hippocampal networks in mouse models of mental illness. Eur J Neurosci.

[42] Aizer A, Stroud L, Buka S (2016). Maternal stress and child outcomes: Evidence from siblings. J Hum Resour.

[43] DiPietro JA (2012). Maternal stress in pregnancy: considerations for fetal development. J Adolesc Heal.

[44] Chang G (2020). Maternal substance use: consequences, identification, and interventions. Alcohol Res.

[45] Newnham JP, Moss TJM, Nitsos I, Sloboda DM, Challis JRG (2002). Nutrition and the early origins of adult disease. Asia Pac J Clin Nutr.

[46] Taylor PD, Poston L (2007). Developmental programming of obesity in mammals. Exp Physiol.

[47] Szutorisz H, Hurd YL (2016). Epigenetic effects of Cannabis exposure. Biol Psychiatry.

[48] Markunas CA, Hancock DB, Xu Z, Quach BC, Fang F, Sandler DP (2021). Epigenome-wide analysis uncovers a blood-based DNA methylation biomarker of lifetime cannabis use. Am J Med Genet Part B Neuropsychiatr Genet.

[49] Hinckley JD, Saba L, Raymond K, Bartels K, Klawitter J, Christians U, et al. An approach to biomarker discovery of cannabis use utilizing proteomic, metabolomic, and lipidomic analyses. Cannabis Cannabinoid Res. 2020; 10.1089/can.2020.0002 PMC8864439 33998853

[50] Zuurman L, Ippel AE, Moin E, Van Gerven JMA (2009). Biomarkers for the effects of cannabis and THC in healthy volunteers. Br J Clin Pharmacol.

[51] Karthiya R, Wasil SM, Khandelia P (2020). Emerging role of N4-acetylcytidine modification of RNA in gene regulation and cellular functions. Mol Biol Rep.

[52] Jin G, Xu M, Zou M, Duan S (2020). The processing, gene regulation, biological functions, and clinical relevance of N4-acetylcytidine on RNA: a systematic review. Mol Ther Acids.

[53] Battistella G, Fornari E, Annoni J-M, Chtioui H, Dao K, Fabritius M (2014). Long-term effects of cannabis on brain structure. Neuropsychopharmacology.

[54] Scheffler F, Du Plessis S, Asmal L, Kilian S, Phahladira L, Luckhoff HK (2021). Cannabis use and hippocampal subfield volumes in males with a first episode of a schizophrenia spectrum disorder and healthy controls. Schizophr Res.

[55] Batalla A, Bhattacharyya S, Yuecel M, Fusar-Poli P, Crippa JA, Nogue S (2013). Structural and functional imaging studies in chronic cannabis users: a systematic review of adolescent and adult findings. PLoS ONE.

[56] Schacht JP, Hutchison KE, Filbey FM (2012). Associations between cannabinoid receptor-1 (CNR1) variation and hippocampus and amygdala volumes in heavy cannabis users. Neuropsychopharmacology.

[57] French L, Gray C, Leonard G, Perron M, Pike GB, Richer L (2015). Early cannabis use, polygenic risk score for schizophrenia and brain maturation in adolescence. JAMA Psychiat.

[58] Cheetham A, Allen NB, Whittle S, Simmons JG, Yücel M, Lubman DI (2012). Orbitofrontal volumes in early adolescence predict initiation of cannabis use: a 4-year longitudinal and prospective study. Biol Psychiatry.

[59] Bloomfield MAP, Hindocha C, Green SF, Wall MB, Lees R, Petrilli K (2019). The neuropsychopharmacology of cannabis: a review of human imaging studies. Pharmacol Ther.

[60] Ferland J-MN, Hurd YL (2020). Deconstructing the neurobiology of cannabis use disorder. Nat Neurosci.

[61] Volkow ND, Swanson JM, Evins AE, DeLisi LE, Meier MH, Gonzalez R (2016). Effects of cannabis use on human behavior, including cognition, motivation, and psychosis: a review. JAMA Psychiat.

[62] Ducci F, Goldman D (2012). The genetic basis of addictive disorders. Psychiatr Clin.

[63] Deak JD, Johnson EC. Genetics of substance use disorders: a review. Psychol Med. 2021;1–12. 10.1017/S0033291721000969 PMC8477224 33879270

[64] Prom-Wormley EC, Ebejer J, Dick DM, Bowers MS (2017). The genetic epidemiology of substance use disorder: a review. Drug Alcohol Depend.

[65] Zhou H, Sealock JM, Sanchez-Roige S, Clarke T-K, Levey DF, Cheng Z (2020). Genome-wide meta-analysis of problematic alcohol use in 435,563 individuals yields insights into biology and relationships with other traits. Nat Neurosci.

[66] Onaivi ES, Ishiguro H, Sgro S, Leonard CM (2013). Cannabinoid receptor gene variations in drug addiction and neuropsychiatric disorders. J Drug Alcohol Res.

[67] Marco EM, García-Gutiérrez MS, Bermúdez-Silva F-J, Moreira F, Guimarães F, Manzanares J (2011). Endocannabinoid system and psychiatry: in search of a neurobiological basis for detrimental and potential therapeutic effects. Front Behav Neurosci.

[68] Gage SH, Jones HJ, Burgess S, Bowden J, Davey Smith G, Zammit S (2017). Assessing causality in associations between cannabis use and schizophrenia risk: a two-sample Mendelian randomization study. Psychol Med.

[69] Vaucher J, Keating BJ, Lasserre AM, Gan W, Lyall DM, Ward J (2018). Cannabis use and risk of schizophrenia: a Mendelian randomization study. Mol Psychiatry.

[70] Johnson EC, Hatoum AS, Deak JD, Polimanti R, Murray RM, Edenberg HJ, et al. The relationship between cannabis and schizophrenia: a genetically informed perspective. Addiction. 2021; 10.1111/add.15534 PMC8492483 33950550

[71] Treur JL, Demontis D, Smith GD, Sallis H, Richardson TG, Wiers RW (2021). Investigating causality between liability to ADHD and substance use, and liability to substance use and ADHD risk, using Mendelian randomization. Addict Biol.

[72] Artigas MS, Sánchez-Mora C, Rovira P, Richarte V, Garcia-Martínez I, Pagerols M (2020). Attention-deficit/hyperactivity disorder and lifetime cannabis use: genetic overlap and causality. Mol Psychiatry.

